# Orbital Onset: The Intricate Journey From Ear Abscess to Cavernous Sinus Thrombosis in a Diabetic Male

**DOI:** 10.7759/cureus.48922

**Published:** 2023-11-16

**Authors:** Osatohanmwen Ekomwereren, Vyshnavidevi Sunkara, Han Grezenko, Yusra H Hamid, Nuzhat Faran, Muhammad Abubakar

**Affiliations:** 1 Trauma and Orthopaedics, Royal Shrewsbury Hospital, Shrewsbury and Telford Hospital NHS Trust, Shrewsbury, GBR; 2 Internal Medicine, Katuri Medical College and Hospital, Guntur, IND; 3 Medicine and Surgery, Guangxi Medical University, Nanning, CHN; 4 Translational Neuroscience, Barrow Neurological Institute, Phoenix, USA; 5 Community Medicine, University of Khartoum Faculty of Medicine, Khartoum, SDN; 6 Internal Medicine, Fatima Memorial Hospital, Lahore, PAK; 7 Internal Medicine, Wah Medical College, Rawalpindi, PAK

**Keywords:** diabetes type 2, complication of treatment, home remedy, peri-orbital cellulitis, septic cavernous sinus thrombosis

## Abstract

Cavernous sinus thrombosis (CST) is a rare, yet severe condition often linked to infections in the nasal and facial areas. We present a case of a 43-year-old male farmer with diabetes who initially showed ear abscess symptoms that progressed to vision loss and CST-like symptoms. Self-treatment and an unidentified medication regimen may have worsened his condition. Advanced diagnostic evaluations, particularly magnetic resonance imaging with magnetic resonance venography, confirmed CST, likely originating from the ear infection spreading to the eyes, causing bilateral orbital cellulitis. Treatment with antibiotics, anticoagulants, and supportive therapy stabilized the patient's condition. This case emphasizes the importance of early detection and intervention in CST, especially in atypical presentations, and the need for comprehensive diagnostic and therapeutic approaches.

## Introduction

The cavernous sinuses, crucial venous structures flanking the sella turcica at the skull's base, can become the epicenter of cavernous sinus thrombosis (CST), a severe and relatively rare medical condition [1]. CST often arises as a complication of infections, notably in the nasal and facial areas, where infections can trigger clot formation within these sinuses. Common conditions predisposing individuals to CST include sphenoiditis, ethmoiditis, facial abscesses, cellulitis, and ocular infections [2]. Despite its potential severity, CST remains infrequent, with an annual diagnosis rate of only 4.5 in 1,000,000 individuals [3].

The objective of this case report is twofold: first, to highlight the profound association between complicated ear and eye infections and the onset of CST, and second, to explore the comprehensive management of this formidable condition. It is pivotal to underscore that while CST might be rare, each unique case, such as the one presented here, contributes significantly to the medical literature. The nuanced understanding, diagnosis, and management strategies from individual case studies like this provide invaluable insights, enriching collective knowledge and guiding clinicians in similar future scenarios.

## Case presentation

A 43-year-old male farmer with a history of diabetes presented to our hospital with concerning symptoms, including a persistent right ear discharge lasting 1.5 months, a month-long fever, and 20-25 days of right eye swelling. Initially, he self-diagnosed an abscess in his right ear canal and attempted home remedies, which worsened the condition. Over time, the infection produced a distinctive yellowish, foul-smelling discharge with a sticky consistency, accompanied by severe pain radiating toward his right eye. This pain was exacerbated by physical activity and did not respond to over-the-counter medications.

Despite seeking local medical advice and receiving an unspecified prescription, his health deteriorated. Approximately two weeks after the onset of ear discharge, he developed intermittent mild fevers, briefly alleviated by medication. However, his condition worsened, and within four to five days, he experienced intense right eye pain, resulting in rapid vision loss. This alarming development was accompanied by persistent, severe headaches, and his left eye exhibited symptoms, primarily blurred vision, with noticeable bulging in both eyes. During our facility's examination, the patient, with a history of diabetes, displayed evident distress, including diminished consciousness, a generally unwell appearance marked by fever and anemia, chemosis in both eyes, and noticeable facial deviation to the right due to the right-sided infection's tightness.

To gain deeper insights into his condition, we conducted diagnostic tests. The results, notably the inflammatory markers, revealed an ongoing inflammatory process. Elevated eosinophil counts indicated an active infection, while increased platelet counts suggested a potential thrombotic event. Table 1 details the initial blood workup.

**Table 1:** Initial complete blood workup of the patient INR: international normalized ratio, APTT: activated partial thromboplastin time, WBC: white blood cell, RBC: red blood cell, HCT: hematocrit, ALT: alanine transaminase, AST: aspartate aminotransferase, ALP: alkaline phosphatase, ESR: erythrocyte sedimentation rate

Coagulation profile		
Specific tests	Results	Reference range
Prothrombin time-control	12	10-14 seconds
Prothrombin time-patient	13	Up to 13 seconds
INR	1.17	0.9-1.3
Control time	34	25-35 seconds
APTT	32	Up to 31 seconds
Hemogram		
WBC count	28.71	4-11 x10^9^/L
Total RBCs	3.2	3.8-5.2 x10^12^/L
Hemoglobin	9.60	13-18 (g/dL)
HCT	30.70	35%-46%
Platelets	586	150-400 x10^9^/L
Neutrophils	77.70	40%-80%
Lymphocytes	15.60	20%-40%
Renal function tests		
Urea	29	10-50 mg/dL
Serum creatinine	0.56	0.5-0.9 mg/dL
Liver function tests		
Bilirubin total	1.42	0.3-1.2 mg/dL
ALT	39	Up to 40 U/L
AST	42	Up to 40 U/L
ALP	219	40-120 U/L
Inflammatory markers		
ESR	37	0-25 mm/1st hour
C-reactive protein quantitative	96	<5

**Figure 1:**
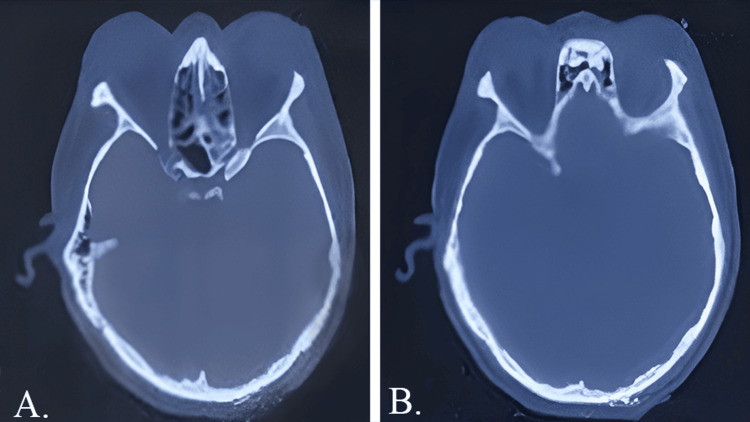
A CT scan of the head shows bilateral otomastoiditis and sinusitis that affected the bilateral maxillary, sphenoid, and ethmoid sinuses, as shown in panels A and B. CT: computed tomography

Given the patient's detailed history and baseline test results, our preliminary hypothesis was that the ear infection had potentially spread to the eye through the orbital sinuses, leading to vision complications. We ordered a computed tomography (CT) scan of the temporal bone to validate this theory. The findings were significant. They revealed bilateral otomastoiditis and sinusitis that affected the bilateral maxillary, sphenoid, and ethmoid sinuses. Most notably, there were clear indications of bilateral orbital cellulitis. Details of the CT scan are provided in Figure 1.

**Figure 2:**
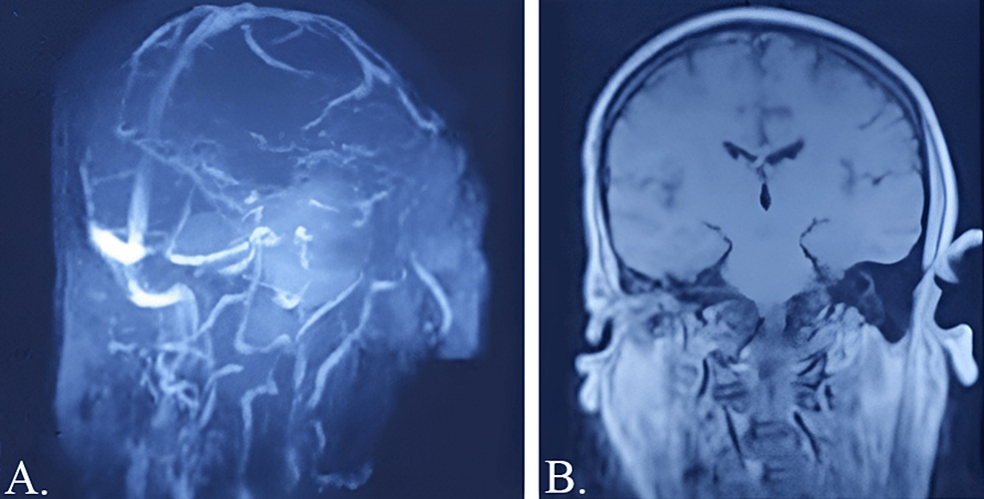
(A) MRV and (B) MRI of the brain. A partial filling defect in the right sigmoid sinus is evident in (A). Extensive abnormal signals in the right orbit involving orbital muscle and optic nerve associated with abnormal signal intensity in the central retrobulbar area and significant edematous changes in periorbital and preseptal soft tissue were found. MRV: magnetic resonance venography, MR: magnetic resonance imaging

To further elucidate the nature and extent of the condition, we proceeded with magnetic resonance imaging (MRI) of the brain with intravenous (IV) contrast. The results were revealing. They showcased the presence of fluid in the right mastoid ear cells, suggestive of mastoiditis, and a partial central filling defect in the right sigmoid sinus, raising suspicions of a thrombus. The MRI also highlighted significant right proptosis with an irregularly shaped right eyeball and extensive abnormal signals within the right orbit. In conjunction with others, these findings strengthened the suspicion of an extensive infectious or inflammatory disease process. Details of the MRI are provided in Figure 2.

Piecing together the clinical, radiological, and imaging data, we concluded that the ear infection had spread to the eyes, leading to bilateral orbital cellulitis. This, in turn, had cascaded to the cavernous sinus, resulting in thrombosis.

The patient received medical management due to the absence of a brain abscess or empyema, making surgery unnecessary. Our multifaceted approach focuses on diabetes control, antibiotics, and anticoagulants. Currently, the patient is on a combination of medications, including Clexane, warfarin, insulin, empagliflozin, meropenem, metronidazole, and topical eye treatments. Along with supportive therapy, this regimen stabilized the patient. Plans for surgical eye decompression are considered when the patient's overall health improves.

## Discussion

Cavernous sinus thrombosis presents a spectrum of symptoms, often reflecting the condition's impact on the cavernous sinuses and their surrounding structures. Established signs of CST encompass headaches, fever, visual disturbances, chemosis, and altered mental states [4]. In the context of our patient, he manifested several of these classic symptoms: fever, vision loss, chemosis, severe headache, and, ultimately, an altered state of consciousness.

CST is a relatively infrequent clinical entity, with an incidence rate of two to five cases per one million individuals. Notably, its prevalence is higher in females and generally surfaces after the age of 40 [5]. Considering our patient's demographics, a male over 40, this presentation is uncommon and warrants special attention in the clinical literature.

Here, we have reported a distinctive manifestation of CST in a diabetic individual, emphasizing the intersection of this rare condition with diabetes mellitus. While CST is a relatively uncommon clinical entity, its occurrence in conjunction with diabetes introduces an intriguing dimension to our understanding of the disease spectrum. Diabetes influences the immune response and vascular dynamics, potentially predisposing individuals to complications from infections, including those originating in the ear [6]. The interplay between diabetes and CST remains an underexplored area in the literature, and our case contributes to bridging this gap. Previous studies have suggested that diabetes can impact the severity and progression of infections, and in the context of CST, it may influence the thrombotic processes within the cavernous sinuses [7]. As diabetic patients often exhibit altered coagulation profiles and impaired immune function, these factors could contribute to the complexity and severity of CST in this population [8]. Further research is warranted to elucidate the mechanisms underlying the interaction between diabetes and CST, with implications for tailored diagnostic and therapeutic approaches in diabetic individuals presenting with symptoms of ear infections progressing to CST.

For diagnostic clarity in suspected CST cases, MRI, complemented with magnetic resonance venography (MRV), has emerged as an invaluable tool, owing to its high sensitivity in detecting thrombosis within the cavernous sinus [9]. Consistent with this protocol, our patient underwent an MRI with MRV, which strongly indicated the presence of cavernous sinus thrombosis.

The management of CST is multifaceted. Standard care involves the administration of intravenous broad-spectrum antibiotics, anticoagulation (commonly with heparin), pain management, hydration, and dedicated ophthalmic care. Hospitalization is imperative; patients are typically advised to keep their heads elevated. Vigilant monitoring is crucial to promptly detect potential complications such as clot extension, stroke, vision loss, or the onset of sepsis [10]. Aligning with this treatment paradigm, our patient received the recommended care and is currently stable. As we chart the way forward, a comprehensive long-term plan will be devised to mitigate systemic complications and optimize patient outcomes.

## Conclusions

Our case report presents an unusual occurrence of cavernous sinus thrombosis in a 43-year-old male, a demographic rarely associated with this condition. Emerging from an ear infection and progressing to bilateral orbital cellulitis, it underscores the rapid spread of infections within interconnected facial structures, highlighting the risks of delayed medical interventions. The vital role of advanced diagnostic tools, particularly MRI with MRV, becomes evident. This case underscores the need for a vigilant clinical approach, as seemingly benign symptoms like headaches combined with eye or ear issues can indicate serious intracranial complications. Timely imaging and aggressive multidisciplinary treatment are imperative to prevent catastrophic outcomes, including clot propagation, vision loss, neurological deficits, and potential fatality. Despite its rarity, CST remains a formidable clinical challenge, demanding sharp clinical judgment and proactive medical care.
